# Visual acuity improvement after treatment of central retinal artery occlusion: a case report

**DOI:** 10.11604/pamj.2024.49.18.44717

**Published:** 2024-09-19

**Authors:** Muhammad Indra Mahardika Iridika Humeri, Ima Yustiarini, Ady Dwi Prakosa, Sauli Ari Widjaja, Muhammad Firmansjah, Wimbo Sasono

**Affiliations:** 1 Department of Ophthalmology, Faculty of Medicine Airlangga University, Dr. Soetomo General Academic Hospital Surabaya, Surabaya, Indonesia

**Keywords:** Central retinal artery occlusion, ophthalmic emergency, ocular massage, acetazolamide therapy, case report

## Abstract

Central Retinal Artery Occlusion (CRAO) is a serious ophthalmic emergency characterized by sudden, painless vision loss in one eye. This condition leads to rapid and significant visual impairment if not treated promptly. This case illustrates an adult man with hypertension presented with unilateral, painless, sudden vision loss occurring 13 hours before admission. Examination revealed a visual acuity of 1-meter counting finger in the affected eye, a cherry red spot, and a pale retina. Diagnosed with CRAO, immediate interventions included ocular massage, and acetazolamide loading alongside systemic antihypertensive medication. Visual acuity improved significantly, with the patient able to see 5/30 on the nasal side and can maintain this visual acuity until 6 months follow-up. Immediate and aggressive treatment for CRAO can lead to significant visual recovery even when initiated beyond the traditionally recommended time frame, underscoring the need for quick recognition and intervention in CRAO cases.

## Introduction

Central Retinal Artery Occlusion (CRAO) is an occlusion that occurs in the central retinal artery, resulting in retinal hypoperfusion, rapidly progressive cellular damage, and vision loss. The most common symptoms of CRAO are unilateral, painless, sudden vision loss. CRAO is an ophthalmic emergency because it will deplete the inner retina from its blood supply, creating retinal ischemia, which further causes vision loss [1]. Despite the rare occasion, less than 20% of patients will regain their visual acuity in the affected eye. Over three-quarters of the affected eye will have a visual acuity of less than 20/400 [2]. CRAO has the best vision prognosis when treated below 90 minutes up to 4 hours after presentation [3]. Ocular massage using three-mirror Goldmann for 20 minutes or anterior chamber paracentesis is helpful to dislodge the thrombus or embolus. Systemic oral acetazolamide could also be given to lower the intraocular pressure to speed up the dislodgment of the embolus further. CRAO must treated as a systemic disease; treating comorbidities such as hypertension, dyslipidemia, and arrhythmia is essential and lifesaving. Collaboration with the cardiologist, internist, and neurologist is mandatory [1,2]. This case report presents a case of a 47-year-old man with uncontrolled hypertension who suffered sudden vision loss 13 hours before presentation at the emergency ward. Despite the presentation to the emergency ward already being past the golden period, the immediate and aggressive therapy produced favorable outcomes.

## Patient and observation

**Patient information:** a 47-year-old man comes to the emergency room with a chief complaint of sudden vision loss in his right eye 13 hours before he presents. He described the sudden vision loss as only being able to see a dot of light in the distance. The patient did not feel any pain, floaters, photopsia, or visual field defect before or during his vision loss. There was no history of trauma or consuming medication or alcohol before these symptoms occurred. The patient had a history of uncontrolled hypertension and only took medication occasionally.

**Figure 1:**
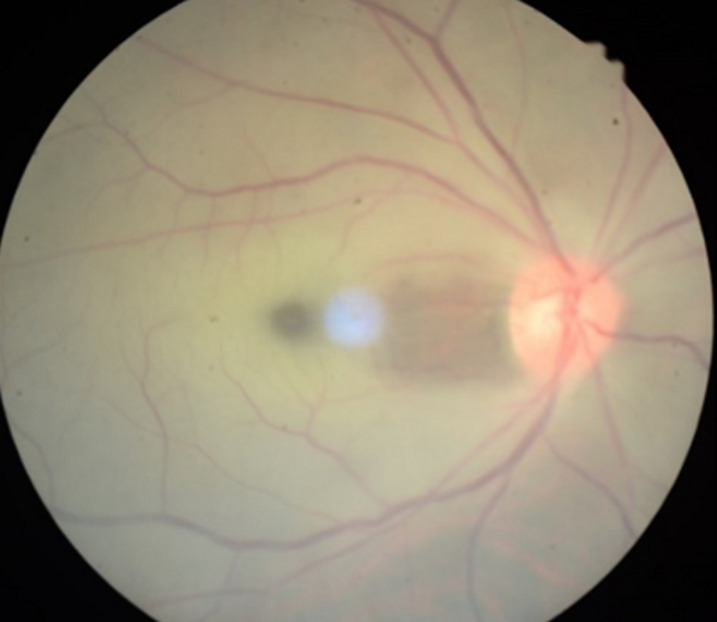
diffuse pale retina, cherry red spot and cilioretinal artery sparring four days after the onset of central retinal artery occlusion at first visit

**Figure 2:**
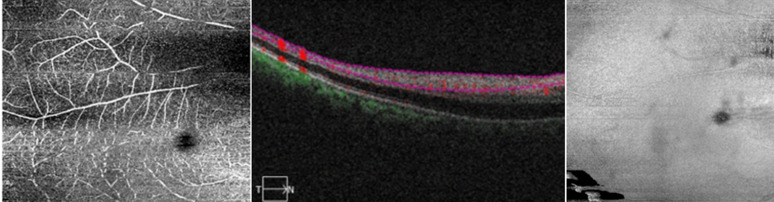
optical coherence tomography angiography examination taken four days after the onset of central retinal artery occlusion shows decreased arterial perfusion in the superficial plexus

**Clinical findings:** the initial visual acuity in his right eye is 3 meters, counting fingers on the temporal side, while the left visual acuity was 6/25. The blood pressure is 220/120. The intraocular pressure is 17 mmHg in RE and 15 in the left eye. There are no other abnormalities in the anterior segment except for minimal lens opacification. We found grade 2 RAPD in his right eye. The posterior segment of the left eye appeared normal. The right eye posterior segment has a diffuse pale retina with a cherry-red spot without retinal artery pulsation and cilioretinal artery sparring (Figure 1, Figure 2).

As a result, we diagnosed the patient with CRAO. Goldmann three-mirror lens was used to perform ocular massage. After 20 minutes of ocular massage, the retinal artery pulse returned. The patient was initially administered 500 mg of acetazolamide, followed by 250 mg thrice daily. A cardiologist was consulted to address the hypertension emergency, while a neurologist was called in to evaluate any neurological abnormalities. No neurological abnormalities were found, and neurotropic vitamins were prescribed.

On the second day of treatment, the patient's visual acuity had not changed. The Relative Afferent Pupillary Defect (RAPD) was still present in the right eye, and the cherry red spot with cilioretinal artery sparing in the posterior segment persisted. On the third day, the visual acuity in the right eye was 5/30 on the nasal side, and the patient was discharged. During the six-month follow-up, the patient could maintain this visual acuity.

**Figure 3:**
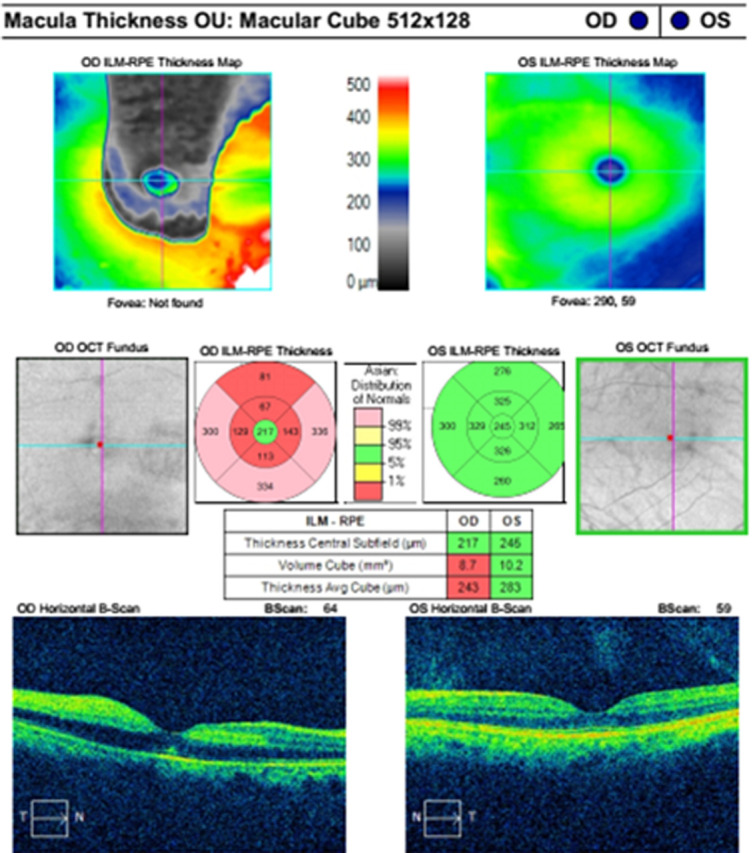
macular optical coherence tomography angiography examination took four days after the onset of central retinal artery occlusion, shows extensive internal limiting membrane-retinal pigment epithelium thinning

**Diagnostic assessment:** the funduscopic examination revealed a diffuse pale retina with a cherry-red spot and cilioretinal artery sparring (Figure 1). There is decreased arterial perfusion in the superficial plexus on Optical Coherence Tomography (OCT)-Angiography (Figure 2) and extensive ILM-RPE thinning (Figure 3).

**Diagnosis:** Central Retinal Artery Occlusion (CRAO).

**Therapeutic interventions:** the patient received ocular massage using a Goldmann three-mirror lens for 20 minutes without undergoing anterior chamber paracentesis due to the presence of retinal artery pulsation post-massage. A 500 mg dose of acetazolamide was administered initially, followed by 250 mg of acetazolamide three times a day for three days to reduce intraocular pressure. Neurotropic vitamins were prescribed to improve vision. Consultations were sought with a cardiologist to address the hypertension crisis and a neurologist to evaluate for potential neurological abnormalities, which were not found. Aggressive systemic hypertension therapy was initiated to manage the blood pressure.

**Patient perspective:** *"I feel so frightened when my vision is suddenly lost. I thought I would not be able to see it again. Even when my vision can´t fully recover like before, I´m grateful for the vision I can regain."*

**Informed consent:** the patient and family were informed of the case reported and agreed that it should be published for the benefit of people and medicine.

## Discussion

CRAO is caused by a blockage in the central retinal artery, which compromises the blood flow from the central retinal artery to the inner layer of the retina. CRAO is believed to occur due to embolic or thrombotic occlusion of the central retinal artery, which results in retinal ischemia. Plaques in the carotid artery and those originating from the heart are considered the primary causes of emboli. Most of these plaques are cholesterol plaques [1]. The prevalence of CRAO itself is rare, ranging from 1.8 to 5.97 per 100.000 people every year. Despite the rare occasion, less than 20% of patients will regain their visual acuity in the affected eye. In 80% of the eyes of 260 individuals with CRAO, the Best-Corrected Visual Acuity (BCVA) was 20/400 or worse. About 10% of people with CRAO have a normal central vision [2,4]. Brown *et al*. studied visual acuity in eyes with CRAO. They reported that 69 of 73 (95%) showed acuity equal to or worse than counting fingers at the time of presentation, and 40 of 60 eyes (66%) showed the same level of final acuity [5]. Patients with Central Retinal Artery Occlusion (CRAO) typically experience sudden, one-sided vision loss that is painless, often resulting in the ability only to perceive hand motions or worse. Their intraocular pressures, anterior chamber examination, and extraocular eye movements are all within normal range. In 15-30% of cases where the cilioretinal artery spares, the visual acuity may be preserved at 20/50 or better, with only peripheral vision being affected [1,6]. Our patient presented with sudden painless vision loss, with a visual acuity of counting fingers from 1 meter, normal intraocular pressure, anterior segment, and extraocular eye movements. Our patient was found to have cilioretinal artery sparing, which prevented total blindness.

It is essential to perform a thorough fundoscopic examination to diagnose CRAO accurately. All patients presenting with symptoms suggestive of CRAO should undergo a dilated fundoscopic examination. During the examination, the retina will appear diffusely pale with a cherry-red spot, a distinctive feature attributed to preserving choroidal circulation beneath the narrow fovea. It is also important to observe any narrowing, “boxcarring,” or segmentation of the arterioles, and the potential visibility of arteriolar emboli. Cilioretinal artery sparing can be observed in approximately 30% of the population as an arcade of an artery spreading from the optic nerve into the macula. In CRAO, cilioretinal artery sparing may appear as a “greyish” area between the optic nerve and macula against a pale background of the retina [1].

CRAO has the best visual prognosis when treated during 90 minutes to 4 hours after the onset [3,7]. Ocular massage using a Goldmann three-mirror lens is the simplest and quickest initial treatment. Goldmann three-mirror lens also enables direct artery viewing, which is necessary for assessing the pulsing of the central retinal artery. The objective is to collapse the arterial lumen mechanically, induce rapid fluctuations in arterial flow, enhance perfusion, and create Intraocular Pressure (IOP) fluctuation to dislodge the embolus or thrombus. One of the described methods consists of applying pressure for 10-15 seconds, followed by a release for 3-5 minutes, and in some literature, up to 20 minutes. The patient can continue self-massaging through their closed eyelids [1,8]. Anterior chamber paracentesis is hypothesized to promote retinal perfusion, particularly in the first several hours of CRAO. This method was performed by withdrawing 0.1-0.2 ml of aqueous humor using a 27-gauge needle. This therapy is controversial but has been advocated by some authorities, and when performed by professionals, this treatment has no complications. On some occasions, a lens injury resulting in a cataract may occur. Ocular massage should be avoided following paracentesis [1,9]. Reducing IOP could aid in dislodging the thrombus. Glaucoma medication such as topical apraclonidine 1%, timolol 0.5%, and intravenous acetazolamide 500 mg induce a more prolonged reduction in intraocular pressure. Hyperosmotic drugs such as mannitol or glycerol have been utilized due to their potentially more immediate IOP-lowering action and increased intravascular volume. Alternatively, a loading dose of acetazolamide could be utilized [1]. Transluminal Nd: YAG laser embolysis/embolectomy has been recommended for Branch Retinal Artery Occlusion (BRAO) or CRAO in a visible occluding embolus (Hollenhorst plaque). Using a fundus contact lens, a configurable number of shots of 0.5-1 mJ or more (up to 2.4 mJ) are applied directly to the embolus. Although expertise with this procedure is limited, the claimed final visual effects are awe-inspiring. The most common complications are subretinal and vitreous hemorrhage, which can be stopped by applying pressure to the globe. The laser embolectomy should not be performed if no visible occluding embolus is found [1,2].

The therapy applied to our patient is ocular massage, using a Goldmann three-mirror lens for 20 minutes. The anterior chamber paracentesis was not performed. A loading dose of 500 mg acetazolamide and a maintenance dose of acetazolamide 250 mg three times a day were used to lower the IOP. The neurotropic vitamin is also administered to improve the vision [10]. A cardiologist was consulted to manage the hypertension crisis, and a neurologist was to observe the neurological abnormalities that were not found in this patient. Aggressive systemic hypertension therapy is prescribed to maintain the blood pressure to a normal level. Despite the rare occasion, less than 20% of patients will regain their visual acuity in the affected eye [1]. Over three-quarters of the affected eye will have a visual acuity of less than 20/400. CRAO has the best vision prognosis when treated below 90 minutes up to 4 hours after presentation. Our patient regained his visual acuity from 1 meter counting fingers to 5/30, but this visual acuity is limited to the nasal side. The presence of cilioretinal artery sparing in this patient prevents the patient from total blindness.

## Conclusion

CRAO is an ophthalmic emergency caused by a central retinal artery occlusion. The main symptoms are sudden unilateral vision loss without pain, a cherry-red spot, and a pale, diffuse retina. CRAO is reversible if treated within 90 minutes to 4 hours before onset, which is the golden window for this condition. If the patient has passed the golden period, visual acuity may be lost entirely, primarily if cilioretinal artery sparing was not performed. The primary treatment for CRAO consists of ocular massage using a Goldmann three-mirror lens to dislodge the clot and acetazolamide loading to reduce intraocular pressure. If there are no signs of the central retinal artery pulsation returning, an anterior chamber aqueous humor paracentesis can be performed. In conjunction with the ophthalmologist's treatment, comorbidities such as hypertension, dyslipidemia, and diabetes must be managed. Consultation with a neurologist, cardiologist, and internist is necessary to maximize the therapeutic effect.
